# Quercetin Alleviates Cerebral Ischemia-Induced Neuroinflammation by Inhibiting Microglia-Mediated NLRP3/Caspase-1/GSDMD Pathway

**DOI:** 10.3390/cells15060552

**Published:** 2026-03-19

**Authors:** Da Shen, Weiao Kong, Haoke Qiu, Huiling Yuan, Wanyi Wu, Lefan Huang, Zixin Yin, Lisheng Chu, Lijun Ge

**Affiliations:** 1 College of Life Science, Zhejiang Chinese Medical University, Hangzhou 310053, China; 202311115311040@zcmu.edu.cn (D.S.); 202411115311005@zcmu.edu.cn (W.K.);; 2 Key Laboratory of TCM Encephalopathy of Zhejiang Province, Zhejiang Chinese Medical University, Hangzhou 310053, China; 3 Department of Physiology, Zhejiang Chinese Medical University, Hangzhou 310053, China

**Keywords:** quercetin, cerebral ischemia, neuroinflammation, pyroptosis, inflammasome

## Abstract

In the pathological cascade of cerebral ischemia, the pyroptosis axis mediated by the NLRP3 inflammasome in activated microglia is a core link driving neuroinflammation and secondary brain injury. Quercetin has been proven to possess multi-target neuroprotective activity, and its anti-inflammatory effect has attracted particular attention. However, direct molecular evidence is lacking regarding how quercetin precisely regulates the NLRP3/Caspase-1/GSDMD core pyroptosis axis in microglia in cerebral ischemia models and whether it can directly target NLRP3 to inhibit this axis, thereby alleviating cerebral ischemic injury. This study aimed to investigate the molecular mechanism by which quercetin alleviates cerebral ischemic injury through inhibiting the pyroptosis axis, combining cellular and animal models with molecular docking and molecular dynamics simulations. The oxygen-glucose deprivation (OGD) model of BV2 microglia and the photothrombotic (PT) model of focal cortical ischemia in male C57BL/6 mice were used to detect the ameliorative effect of quercetin on cerebral ischemia-related injury through cellular and animal experiments. AutoDock Vina 1.5.7 and GROMACS 2025.3 software were employed for molecular docking and molecular dynamics simulations, respectively, to analyze the binding mode and complex stability between quercetin and the NLRP3 protein. The results showed that quercetin could significantly ameliorate OGD-induced injury in BV2 cells and downregulate the expression of pyroptosis and inflammation-related proteins and factors. Meanwhile, it relieved motor dysfunction in PT mice, attenuated cortical neuronal injury, and inhibited the activation of the cerebral pyroptosis axis. At the molecular level, molecular simulation predictions indicated that quercetin might specifically bind to the NACHT domain of the NLRP3 protein, forming a complex with a stable conformation, and van der Waals interactions served as the main driving force for binding. This study confirmed that quercetin can directly bind to the NLRP3 protein and alleviate cerebral ischemia-induced inflammatory injury by inhibiting the activation of the NLRP3/Caspase-1/GSDMD pyroptosis axis and the release of downstream inflammatory factors. Combined with the molecular simulation results, a predictive hypothesis is proposed: direct binding of quercetin to the NLRP3 protein is one of its core mechanisms of action. These findings provide direct experimental evidence for the development of NLRP3-based drugs against ischemic brain injury.

## 1. Introduction

Cerebral ischemia refers to a disease characterized by insufficient blood supply to a certain part of the brain, leading to impaired brain function and potential complications such as cerebral thrombosis, which seriously endangers human life and health [1]. Among various mechanisms, the inflammatory response is an important contributor to cerebral ischemic injury [2,3], making the search for highly effective anti-inflammatory drugs a core direction in the research of cerebral ischemia treatment.

Pyroptosis, a pro-inflammatory form of programmed cell death, has emerged as a critical mediator of secondary brain injury following ischemia. In activated microglia, danger-associated molecular patterns (DAMPs) trigger the assembly of the NOD-like receptor pyrin domain-containing 3 (NLRP3) inflammasome—a multiprotein complex comprising NLRP3, the adaptor ASC, and pro-caspase-1. Upon activation, caspase-1 cleaves gasdermin D (GSDMD) to generate its N-terminal fragment (GSDMD-N), which oligomerizes in the plasma membrane to form pores, causing cell lysis and release of mature IL-1β and IL-18 [4,5,6]. Accumulating evidence indicates that inhibition of the NLRP3/caspase-1/GSDMD pyroptosis axis confers neuroprotection in experimental models of cerebral ischemia [7,8], positioning this pathway as a promising therapeutic target [9].

Quercetin (Que), a natural flavonoid abundant in fruits and vegetables, exhibits potent antioxidant and anti-inflammatory properties. Previous studies have demonstrated that quercetin attenuates cerebral ischemic injury by reducing infarct volume, improving neurological deficits, and preserving blood-brain barrier integrity, potentially via the Sirt1/Nrf2/HO-1 pathway or suppression of apoptotic signaling [10,11,12,13]. Notably, quercetin has been shown to downregulate NLRP3 inflammasome components in various inflammatory models. However, it remains unclear whether quercetin directly targets the NLRP3 protein to inhibit inflammasome assembly and subsequent pyroptosis in microglia during cerebral ischemia. Crucially, direct molecular evidence confirming the binding affinity, binding site, and structural stability of the quercetin–NLRP3 interaction is lacking.

As the resident immune cells of the central nervous system (CNS), microglia are key regulators of the neuroinflammatory cascade after cerebral ischemia/reperfusion (I/R) injury [14]. Under ischemic stimulation, microglia undergo rapid morphological and functional polarization: the proinflammatory M1 phenotype is activated, characterized by excessive secretion of proinflammatory cytokines such as IL-1β, IL-6 and TNF-α and assembly of the NLRP3 inflammasome, ultimately exacerbating neuronal death and blood-brain barrier damage [15]. In contrast, the anti-inflammatory M2 phenotype promotes tissue repair and neuroprotection. Therefore, targeting microglial dysfunction and shifting their polarization balance toward the M2 phenotype is a promising therapeutic strategy for ischemic stroke.

Notably, a growing number of preclinical studies have shown that quercetin is a potent modulator of microglial reactivity. Quercetin has been proven to inhibit the excessive activation of microglia in various CNS disease models, including neuroinflammation, Alzheimer’s disease and traumatic brain injury [16]. Mechanistically, quercetin can inhibit LPS/IFN-γ-induced M1 polarization of microglia and promote the expression of M2-specific markers such as Arg-1 and CD206, thereby alleviating the proinflammatory microenvironment [17]. However, despite these advances, it remains unclear whether quercetin exerts its neuroprotective effect by directly targeting the NLRP3 inflammasome in microglia in the context of cerebral I/R injury. This key scientific gap forms the basis of the present study, which aims to specifically investigate the role of quercetin in regulating NLRP3 activation in microglia and its impact on neuronal survival.

As core tools of computer-aided drug design, molecular docking and molecular dynamics simulation technologies can accurately predict the binding mode, affinity, and structural stability between small-molecule compounds and target proteins at the atomic level, providing direct visual evidence for clarifying drug targets and molecular mechanisms [18,19]. Based on this, this study intends to verify the protective effects of quercetin on cerebral ischemic injury at the cellular and animal levels through the BV2 microglia OGD/R model [20] and the C57BL/6J mouse PT model [21]. Meanwhile, AutoDock Vina software is innovatively introduced to conduct molecular docking experiments to predict the binding sites, binding modes, and affinity between quercetin and the NLRP3 protein. GROMACS software is used for long-term molecular dynamics simulations to analyze the conformational stability, key interaction residues, and binding driving forces of the quercetin-NLRP3 complex, thereby clarifying the targeted binding relationship between quercetin and the NLRP3 protein at the molecular level. This study aims to systematically elucidate the neuroprotective effect and molecular mechanism of quercetin against cerebral ischemic injury through the NLRP3 inflammasome/pyroptosis axis, providing experimental basis and theoretical support for its clinical application and the development of targeted drugs.

## 2. Materials and Methods

### 2.1. Materials

Quercetin (117-39-5) was purchased from Shanghai Jizhi Biochemical Technology Co., Ltd. (Shanghai, China); MCC950 inhibitor (SJ-MX0058B) and PBS (CRO013-500ML) from Shandong Sikejie Biotechnology Co., Ltd. (Jinan, China); Reactive Oxygen Species Assay Kit (S0033S), MTT Cell Proliferation and Cytotoxicity Assay Kit (C0009S), Lactate Dehydrogenase (LDH) Cytotoxicity Assay Kit (C0016), BCA Protein Assay Kit (P0010S), RIPA Lysis Buffer (P0013C) and Protease Inhibitor Cocktail (P1008) from Shanghai Beyotime Biotechnology Co., Ltd. (Shanghai, China); QuantiCyto^®^ Rat IL-18 ELISA kit (ERC010.48), QuantiCyto^®^ Rat IL-1β ELISA kit (ERC007.48) and QuantiCyto^®^ Rat TNF-α ELISA kit (ERC102a.48) from Shenzhen ExCell Biology Co., Ltd. (Shenzhen, China); Hoechst33342 Fluorescent Dye (B8040) from Beijing Solarbio Science & Technology Co., Ltd. (Beijing, China); Fetal Bovine Serum (BS-1105) from Inner Mongolia Opsci Biotechnology Co., Ltd.(Hohhot, China); DMEM High Glucose Medium (C11995500BT), DMEM Glucose-Free Medium (11966-025) and Trypsin-EDTA Solution (25200-056) from Thermo Fisher Scientific Inc. (Waltham, MA, USA); GAPDH (AF7021) and HRP-conjugated goat anti-rabbit IgG antibody (S0001) from Jiangsu Qinke Biological Research Center Co., Ltd. (Liyang, China); NLRP3 (ET1610-93), Caspase-1 (HA721144) and ASC (HA721306) antibodies from Huaan Biotechnology Co., Ltd.(Hangzhou, China); GSDMD (A20728) antibody from Abclonal Technology Co., Ltd.(Wuhan, China); Rose Bengal Stain (330000-5G) from Sigma-Aldrich Co., LLC (Saint Louis, MO, USA).

### 2.2. Experimental Animals

SPF-grade C57BL/6J male mice, aged 6–8 weeks and weighing approximately 20–25 g, were purchased from Shanghai Slack Laboratory Animal Co., Ltd. (Shanghai, China) [Laboratory Animal Production License No.: SCXK (Hu) 2022-0004]. The experimental animals were raised in the Animal Experiment Center of Zhejiang Pharmaceutical University [Laboratory Animal Use License No.: SYXK (Zhe) 2021-0012]. During the feeding period, sufficient food and drinking water were provided, along with a good feeding environment with a 12 h light/12 h dark cycle. All animal experimental protocols were approved on 25 July 2022, by the Animal Ethics Committee (IRB) of Zhejiang Chinese Medical University (Approval Code: IACUC-20220725-06), in compliance with the institutional ethical guidelines for animal research.

### 2.3. Preparation of Photothrombosis Model and Animal Grouping

Fifty C57BL/6J mice were randomly divided into the Sham group, the Stroke group, the Stroke+Que group and the Stroke+MCC950 group. Quercetin (100 mg/kg) and MCC950 (10 mg/kg) were intraperitoneally injected 24 h after modeling. Mice were anesthetized with 1% sodium pentobarbital (70 mg/kg) by intraperitoneal injection and fixed on a stereotaxic apparatus. The head hair was shaved and disinfected with iodophor, the scalp was incised to expose the skull. With the bregma as the origin, the modeling site was located 2 mm to the right of the sagittal suture and 2 mm posterior to the coronal suture (the motor cortex area, corresponding to the primary motor cortex (M1) and secondary motor cortex (M2) areas in anatomy). The skull was polished while dropping sterile normal saline until the cerebral blood vessels could be clearly seen. Rose Bengal (100 mg/kg) was intraperitoneally injected, and 5 min later, the modeling site was irradiated with a cold light source (532 nm) for 20 min. After irradiation, the mouse scalp was disinfected and sutured, and the mice were placed on an electric blanket to maintain the temperature at 37 °C until awakening and then returned to the cage. Seven days after PT model treatment, mice were anesthetized to harvest the whole brain, and the penumbral tissue of the cerebral cortex was taken for subsequent experiments, with the remaining tissue stored in a −80 °C refrigerator. Subsequent behavioral and related detections were completed 24 h after modeling.

### 2.4. Cell Culture

Mouse microglia (BV2) were purchased from the Cell Bank of the Chinese Academy of Sciences. Cells were cultured in complete medium (95% high-glucose DMEM medium +5% fetal bovine serum +0.5% double antibody) and placed in a 37 °C, 5% CO_2_ incubator; antibiotics were added to the medium during culture to prevent bacterial contamination. Before all formal experiments, mycoplasma detection was performed on the cells by PCR, and all results were negative to ensure no mycoplasma contamination. Cells were passaged when they covered 80% of the culture flask area, and all formal experiments were performed using cells in the logarithmic growth phase that were passaged 3–5 times after resuscitation to ensure the stability of cell state and the repeatability of experimental results.

### 2.5. Preparation of OGD/R Model and Grouping

Cells were divided into four groups: normal (Ctrl) group, OGD/R group, OGD/R+quercetin group, and OGD/R+MCC950 inhibitor group. For the OGD/R model group: the culture medium was discarded and the cells were washed 1–2 times with PBS, replaced with glucose-free Eagle’s medium, and the cells were placed in an anoxic device with continuous ventilation of 95% N_2_ and 5% CO_2_ for 5 min, after which the inlet and outlet ports were closed. The anoxic device was placed in a 37 °C constant temperature incubator for 8 h of hypoxia and glucose deprivation, then taken out, added with high-glucose DMEM medium, and returned to the 5% CO_2_ incubator for 16 h of reoxygenation and glucose restoration. For the OGD/R+quercetin group: after 8 h of hypoxia and glucose deprivation, pre-prepared high-glucose DMEM medium containing quercetin was added, and the cells were returned to the 5% CO_2_ incubator for 16 h of reoxygenation and glucose restoration. For the OGD/R+MCC950 inhibitor group: after 8 h of hypoxia and glucose deprivation, pre-prepared high-glucose DMEM medium containing MCC950 inhibitor was added, and the cells were returned to the 5% CO_2_ incubator for 16 h of reoxygenation and glucose restoration.

### 2.6. MTT Assay for Cell Viability

BV2 cells were uniformly seeded in 96-well plates at a density of 1 × 10^4^ cells/well, with 6 replicate wells in each group. After culturing according to the above grouping and reaching a cell density of approximately 70–80%, the culture medium was discarded, the cells were washed 1–2 times with PBS, and subjected to oxygen-glucose deprivation/reoxygenation treatment; 10 μL of MTT culture solution was added to each well and incubated at 37 °C for 4 h; the culture medium was discarded, 100 μL of dimethyl sulfoxide (DMSO) was added to adhere to the wells, and shaken at low speed for 15 min in the dark; the absorbance was measured at a wavelength of 490 nm using a microplate reader (Molecular Devices, Silicon Valley, USA), and the viability value was calculated.

### 2.7. Detection of Intracellular Reactive Oxygen Species (ROS)

BV2 cells were seeded in 96-well plates at a density of 1 × 10^4^ cells/well. After treatment according to the above grouping, the probe was loaded first: DCFH-DA was diluted 1:1000 with serum-free medium to a final concentration of 10 μM, the old medium was removed, 70 μL was added to each well, and incubated for 20 min in the dark; washed 3 times with serum-free medium to fully remove uninternalized DCFH-DA; observed and photographed under a fluorescence inverted microscope (Zeiss Axio Observer.A1, Jena, Germany) for recording. Quantitative analysis of ROS: fluorescence images were quantitatively analyzed using Image J 1.54g software to determine the total fluorescence intensity of each field of view in each group, and the relative total fluorescence intensity of each group was calculated after deducting the background fluorescence intensity, which was used as a quantitative index of intracellular ROS levels.

### 2.8. Detection of Intracellular Lactate Dehydrogenase (LDH)

Cells were seeded in 96-well plates at a density of 1 × 10^4^ cells/well and treated according to the above grouping. The culture medium of each well was transferred to a 1.5 mL centrifuge tube, centrifuged at 1500 r/min for 5 min, and the supernatant was transferred to a new 96-well plate; 30 μL of LDH working solution was added to each well and mixed (prepared according to the kit instructions); shaken at room temperature for 30 min in the dark, and the absorbance was detected at 490 nm using a microplate reader.

### 2.9. Transmission Electron Microscopy (TEM)

After grouping and modeling, BV2 cells were seeded in 10 cm culture dishes at a density of 5 × 10^5^ cells/dish. After grouping and modeling according to 1.3, cells were digested and collected with 0.25% trypsin, centrifuged and the supernatant was discarded, fixed overnight at 4 °C with 2.5% glutaraldehyde fixative, the glutaraldehyde fixative was discarded, washed 3 times with PBS, fixed with 1% osmium tetroxide fixative at room temperature for 2 h in the dark, washed 3 times with PBS, and dehydrated with gradient acetone: 30%, 50%, 70%, 80%, 90% acetone for 15 min each, and 100% acetone twice for 20 min each; after infiltration with acetone-embedding agent mixture for 2 h each, pure EPON812 epoxy resin embedding agent was added and infiltrated at 4 °C overnight; the samples were embedded in molds and polymerized at 37 °C for 12 h, 45 °C for 12 h and 60 °C for 24 h in sequence to complete the preparation of embedding blocks. After solidification, ultrathin sections (70 nm) were cut with an ultramicrotome, double-stained with 3% uranyl acetate-lead citrate, and cell morphology was observed under a Hitachi H-7650 TEM (Hitachi, Tokyo, Japan), and photographed at 10,000 magnification. Three independent cell culture samples were selected in each group, and 5 non-overlapping fields of view were randomly selected for each sample for shooting; after shooting, representative images were screened according to the typicality of cell morphological characteristics, that is, field of view images with >80% pyroptotic/normal morphological characteristics were selected as the images for result display.

### 2.10. Hoechst33342/PI Double Staining

To detect cell pyroptosis, cells were seeded in 6-well plates at a density of 2.5 × 10^5^ cells/well and treated according to the above grouping. 5 μL of Hoechst staining solution and 5 μL of PI staining solution were added to each well and mixed; incubated on ice for 30 min, washed 1–2 times with PBS; observed and photographed under a fluorescence inverted microscope (Axio Observer. A1).

### 2.11. ELISA for Detection of Inflammatory Factor Expression Levels

BV2 cells were seeded in 6-well plates at a density of 2.5 × 10^5^ cells/well and treated according to the above grouping, and the supernatant was collected. Six mice were selected in each group, anesthetized by intraperitoneal injection of 1% sodium pentobarbital (70 mg/kg) for general anesthesia, and blood was collected by enucleation. The blood was left to stand for 30 min, then centrifuged at 3000 r/min at 4 °C for 15 min, and the supernatant was collected. The kit was taken out of the refrigerator and equilibrated at room temperature for 30 min, and operated according to the kit instructions. The microplate reader was turned on 20 min in advance, zeroed with the blank well, and the absorbance (OD value) of each well was measured at a wavelength of 450 nm. The concentrations of IL-1β, IL-18 and TNF-α in each sample were calculated according to the standard curve.

### 2.12. Western Blot Detection

Cells were seeded in 6-well plates at a density of 2.5 × 10^5^ cells/well and treated according to the above grouping, and digested with trypsin for collection. Four mice were selected in each group, anesthetized to harvest the brain, the penumbral tissue of the cerebral cortex was isolated and weighed, and transferred into a 1.5 mL EP tube. Pre-cooled RIPA lysis working solution (RIPA lysis buffer: protease inhibitor: 0.05 M EDTA = 20:1:1) was added, ground and left on ice for 30 min, centrifuged at 12,000 r/min at 4 °C for 5 min, and the supernatant was the total protein extract, which was quantified using a BCA protein quantification kit (Beyotime, Shanghai, China)and denatured in a 95 °C metal bath for 5 min. Protein samples were subjected to 10% or 12% SDS-PAGE gel electrophoresis (concentrating gel at 80 V for 30 min, separating gel at 120 V for 90 min), transferred to a 0.45 μm polyvinylidene fluoride (PVDF) membrane using a wet transfer system (Bio-Rad, Hercules, CA, USA) at 200 mA for 90 min, and blocked with 5% skimmed milk at room temperature for 2 h. The membrane was incubated with primary antibodies against NLRP3 (1:1000), GSDMD (1:1000), ASC (1:1000), Caspase-1 (1:1000) and GAPDH (1:5000) at 4 °C overnight, washed 3 times with TBST, then incubated with HRP-conjugated goat anti-rabbit IgG (1:20,000) at room temperature for 2 h, washed 3 times with TBST again, and protein bands were visualized using a fluorescence and chemiluminescence imaging system (Clinx, Shanghai, China). Protein bands were quantitatively analyzed using Image J software. The number of biological replicates for Western Blot detection in this experiment was set to *n* = 3, and all samples were subjected to simultaneous electrophoresis and blotting to ensure the consistency of experimental conditions; the repeatability of the results had been verified by pre-experiments before the experiment, and strict animal inclusion/exclusion criteria and standardized motor cortex tissue dissection technology were adopted to minimize intra-group variability.

### 2.13. Behavioral Tests

Six mice were randomly selected from each group for behavioral tests. Grid test: Mice were first placed on a horizontal metal grid (12 cm × 12 cm, grid spacing 1 cm), and a camera was placed under the grid to record the limb support during mouse crawling. Observed for 3 min, and recorded the number of times the mouse’s limbs slipped during crawling. The error step rate was calculated as (number of left limb slips/100) × 100%. Cylinder test: Mice were placed in a transparent organic glass cylinder with a size of 10 cm × 15 cm (diameter × height). When the mouse stood in the cylinder, it would use one front limb or both front limbs to support the body on the cylinder wall. A camera was placed above the cylinder to record the wall-adhering situation of the mouse’s front limbs. Observed for 5 min, and recorded the number of times the right limb, left limb, and both limbs adhered to the cylinder wall. The asymmetry rate was calculated as (R − L)/(R + L + B) × 100%, where R = number of right front limb wall-adhering times, L = number of left front limb wall-adhering times, and B = number of both front limbs wall-adhering times.

### 2.14. HE Staining and Nissl Staining

Six mice were selected in each group, anesthetized and decapitated to harvest the brain, fixed in 4% paraformaldehyde for 24 h, then dehydrated and embedded in paraffin. The embedded brain tissue was sectioned into paraffin sections with a thickness of 4 μm. After dewaxing and rehydration, the sections were stained in hematoxylin solution for 5 min, rapidly differentiated with 1% hydrochloric acid alcohol, blued with ammonia water, rinsed with running water for about 1 min, then counterstained in eosin solution for 1–3 min. The sections were dehydrated in gradient alcohol, xylene I and II, mounted with neutral gum, and observed for pathological changes using a digital pathological section scanning system C13210-01 (Hamamatsu, Japan); the sections were stained with 1% toluidine blue and placed in an oven at 60 °C for 30 min. After washing away the excess dye with distilled water, dehydrated in 95% and 100% ethanol, cleared with xylene, mounted with neutral gum, and observed for pathological changes using a digital pathological section scanning system C13210-01.

### 2.15. Immunohistochemical Detection

Paraffin sections of brain tissue from six mice in each group were dewaxed to water by routine methods, rinsed with running water, and immersed in 0.01 mol/L sodium citrate buffer (pH = 6.0) for antigen retrieval by water bath at 60 °C overnight. After 18 h, naturally cooled to room temperature, washed 3 times with PBS, blocked specific immune antigens with 5% goat serum (diluted with PBS). After blocking, NLRP3 primary antibody (1∶100) was added and incubated at 4 °C overnight. On the second day, the sections were rewarmed, washed 3 times with PBS buffer, HRP-conjugated goat anti-rabbit IgG secondary antibody (1:500) was added dropwise and incubated at room temperature for 1 h, washed 3 times with PBS buffer. DAB staining (observed under microscope), rinsed with tap water, counterstained with hematoxylin for about 2–3 min, rinsed with tap water, blued with PBS for 1 min, dehydrated in gradient alcohol of 75%, 95% and 100% concentration, immersed in xylene solution, sealed with neutral gum, and NLRP3-positive cells were observed and counted using a digital pathological section scanning system C13210-01.

### 2.16. Molecular Docking Verification

The three-dimensional structure of the NLRP3 protein was downloaded from the PDB database (https://www.rcsb.org/, accessed on 21 January 2026). PyMOL 3.0.5 software was used to remove water molecules, crystal water, and irrelevant ligands from the structure, retaining the core functional domain of the NLRP3 protein. The protonation state of the protein was optimized using Propka 3.5.1 software to ensure that the dissociation state of amino acid residues meets physiological conditions. Subsequently, AutoDockTools 1.5.7 software was used to add polar hydrogen atoms to the protein, and the Kollman charge assignment scheme was adopted to calculate the overall charge distribution. Finally, the processed protein was saved in PDBQT format as the receptor molecule for molecular docking.

The 2D structure of quercetin was retrieved from the PubChem database (http://pubchem.ncbi.nlm.nih.gov/, accessed on 21 January 2026), and its 3D structure was constructed and energy-minimized using ChemBio2D 22.2 software, then converted to a mol2 format file. The protonation state of the ligand was optimized using Propka software, polar hydrogen atoms were added and Gasteiger charges were assigned via AutoDockTools software, and saved in PDBQT format as the docking ligand. AutoDock Vina software was used for molecular docking experiments, with the NACHT domain of the NLRP3 protein as the docking pocket, and the grid box was set to cover the active center area. After running the docking program, the optimal binding conformation was selected based on the ranking of binding free energy (ΔG), and evaluated according to the binding free energy.

### 2.17. Molecular Dynamics Simulation

Molecular dynamics (MD) simulation of the protein-ligand complex was carried out in the GROMACS 2025.3 software package [22]. The optimal quercetin-NLRP3 complex structure selected by molecular docking was placed in a periodic boundary cubic box, the AMBER14SB force field [23] was used to describe the protein, and the TIP3P water model was used for solvation to ensure that the complex surface was covered with a solvent layer of at least 1.0 nm thickness. The atomic charges and geometric structure of the ligand quercetin were optimized by quantum mechanical (QM) calculations using the ORCA 6.0 program [24,25]: first, full geometric optimization was completed in the gas phase using the B97-3c hybrid density functional theory (DFT) method, then single-point energy calculation was performed using the B3LYP functional combined with D3 dispersion correction and the def2-TZVP basis set, and the RIJCOSX approximation was adopted to balance calculation accuracy and efficiency. The GROMACS topology file of quercetin was generated using Sobtop 1.0 (dev5) software, and 150 mM NaCl was added to the system to simulate physiological ion concentration and neutralize the overall charge of the system. Before simulation, energy minimization was performed (combining the steepest descent method and conjugate gradient method until energy convergence), followed by 100 ps NPT equilibration under position restraint (temperature 300 K, using V-rescale temperature control; pressure 1 bar, using Parrinello-Rahman pressure control), and finally 100 ns production simulation was carried out with an integration step of 2 fs, and trajectory files were saved every 10 ps during the simulation.

Trajectory analysis was performed using GROMACS built-in tools and self-written scripts. First, periodic artifacts were removed and the system was centered. The main analysis indicators included: root mean square deviation (RMSD) of Cα atoms of the protein backbone, radius of gyration (Rg) of the system, root mean square fluctuation (RMSF) of amino acid residues, solvent-accessible surface area (SASA) of the complex, and the number of hydrogen bonds between the protein and ligand. The molecular mechanics Poisson-Boltzmann surface area (MM-PBSA) method was used to calculate the binding free energy (ΔGbind) of the complex, which was decomposed into molecular mechanics interaction energy (ΔEMM, including van der Waals energy ΔEvdW and electrostatic interaction energy ΔEele), polar solvation energy (ΔGPB), and non-polar solvation energy (ΔGSA); through per-residue free energy decomposition analysis, the contribution of each amino acid residue to the binding free energy was quantified, and key “hotspot” residues at the binding interface were identified.

### 2.18. Statistical Analysis

All experimental data were expressed as the mean ± standard deviation (SD) (*n* ≥ 3). Graph Pad Prism 8.0 software was used for statistical analysis by one-way analysis of variance (One-way ANOVA) and Tukey’s multiple comparison test, with *p* < 0.05 considered statistically significant.

## 3. Results

### 3.1. Effects of Different Concentrations of Quercetin on OGD/R Cell Viability

**Figure 1:**
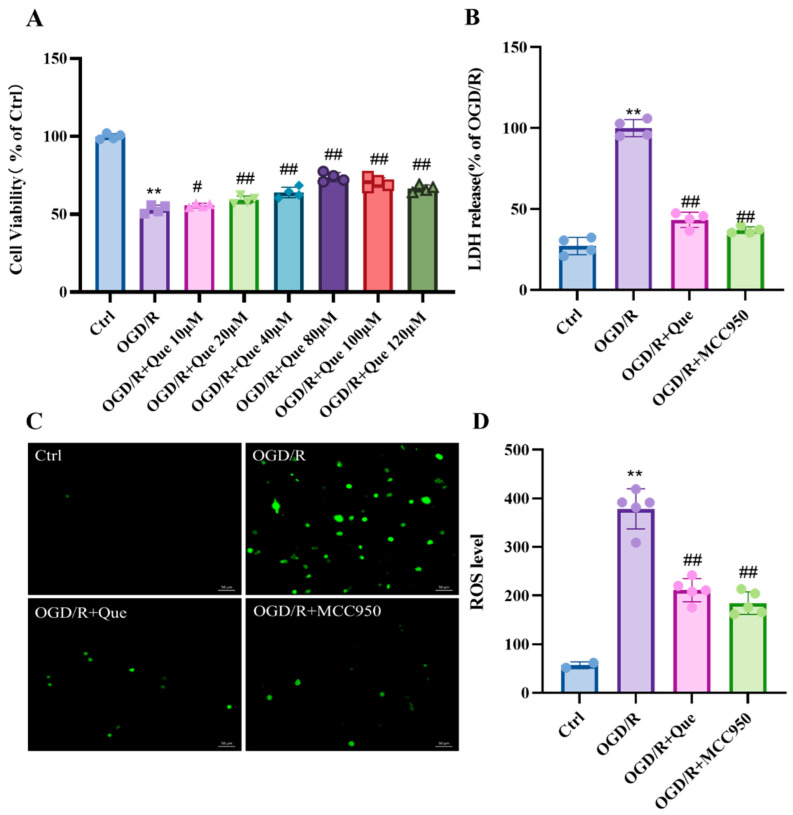
Quercetin inhibits OGD/R-induced microglial injury. (**A**) MTT assay for the survival rate of OGD/R cells treated with different concentrations of quercetin (*n* = 4). (**B**) LDH method for detecting the total amount of lactate dehydrogenase released by cells (*n* = 4). (**C**) Representative images of ROS; scale bar = 50 μm. (**D**) Quantitative analysis of ROS levels (*n* = 5). Compared with the Ctrl group, ** *p* < 0.01; compared with OGD/R group, # *p* < 0.05, ## *p* < 0.01; Mean ± SD, *n* ≥ 3.

Within a certain concentration range, the viability of BV2 cells increased with the increase in quercetin concentration, indicating that quercetin can alleviate OGD/R-induced cell injury. When the concentration of quercetin was 100 μmol/L, the cell viability was significantly increased compared with the OGD/R group, and this concentration was selected for subsequent experiments in this study (Figure 1A).

### 3.2. Quercetin Inhibits OGD/R-Induced Microglial Injury

When the cell membrane ruptures, LDH is released into the extracellular space. Therefore, the integrity of the cell membrane can be inferred by detecting the LDH content in the cell supernatant to evaluate cell pyroptosis. The results showed that compared with the Ctrl group, the LDH content in the OGD/R group was significantly increased, while after quercetin treatment, the LDH content was decreased, and its effect was similar to that of MCC950 (Figure 1B). The increase in intracellular reactive oxygen species is one of the markers of BV2 cell activation, which promotes BV2 cells to secrete more pro-inflammatory factors and exacerbate inflammation. To determine whether quercetin reduces OGD/R-induced reactive oxygen species production, ROS detection was performed. The results showed that the ROS content in the OGD/R group was increased, while quercetin treatment significantly inhibited the production of intracellular reactive oxygen species (Figure 1C,D).

### 3.3. Quercetin Improves OGD/R-Induced Pyroptotic Morphology of BV2 Cells

**Figure 2:**
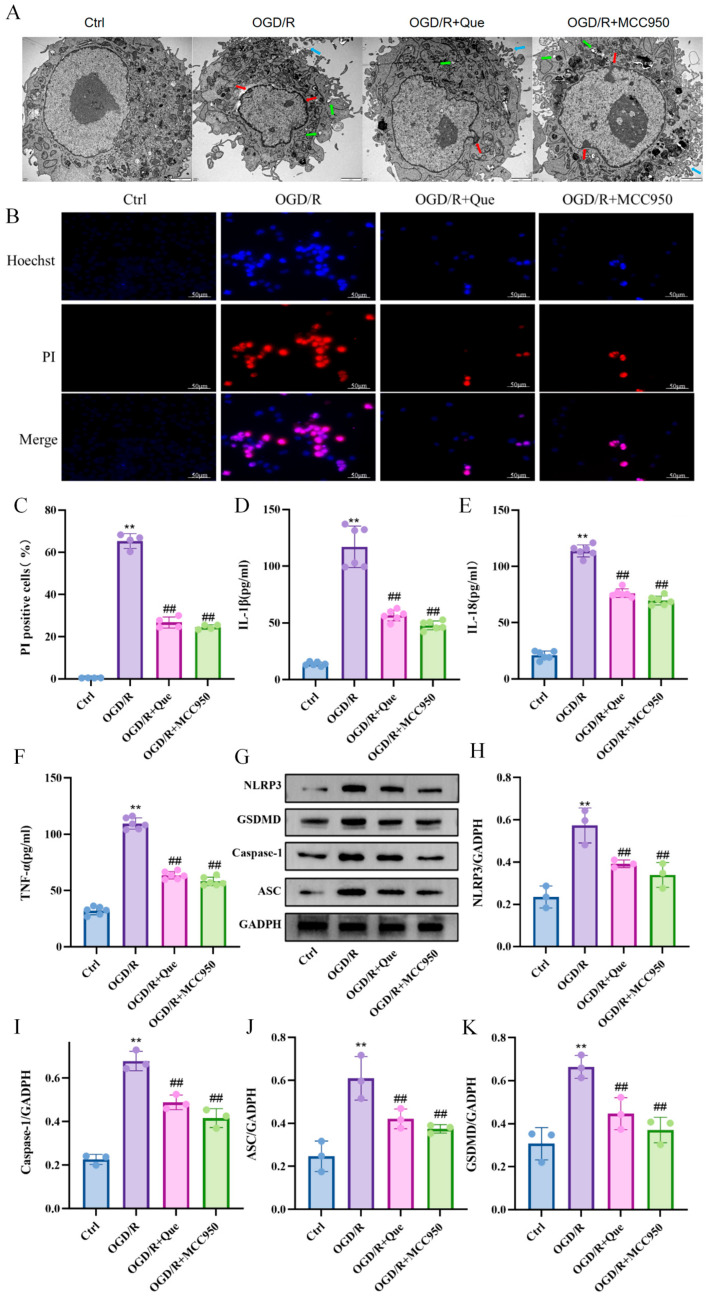
Quercetin alleviates OGD/R-induced cell pyroptosis by inhibiting NLRP3 inflammasome activation to regulate the pyroptosis axis. (**A**) Representative images of transmission electron microscopy, blue arrows indicate cell membrane rupture and content release; red arrows indicate chromatin margination; green arrows indicate intra-membrane vesicles, scale bar = 2 μm. (**B**) Representative images of Hoechst/PI double staining, scale bar = 50 μm. (**C**) Quantitative analysis of Hoechst/PI double staining (*n* = 4). (**D**–**F**) ELISA for detecting the expression levels of IL-1β, IL-18, and TNF-α in the cell supernatant *(n* = 6). (**G**) Representative Western blot images of NLRP3, Caspase-1, ASC, and GSDMD; (**H**–**K**) Quantitative analysis of NLRP3, Caspase-1, ASC, and GSDMD proteins (*n* = 3). Compared with Ctrl group, ** *p* < 0.01; compared with OGD/R group, ## *p* < 0.01; Mean ± SD, *n* ≥ 3.

Transmission electron microscopy was used to observe the morphology of BV2 cells. Compared with the control group, the OGD/R group cells exhibited typical pyroptotic morphology, including intact nuclear membrane, chromatin margination, damaged cell membrane integrity, and obvious vesicular edema in the cytoplasm, while quercetin treatment significantly improved the pyroptotic morphology of the cells (Figure 2A).

### 3.4. Quercetin Inhibits OGD/R-Induced Cell Pyroptosis

Hoechst can penetrate the cell membrane to stain the nucleus, while PI is a nucleic acid dye that cannot stain normal or apoptotic cells but can stain necrotic cells. Therefore, Hoechst/PI double staining was used in this study to verify whether quercetin can inhibit cell pyroptosis. The staining results showed that compared with the Ctrl group, the OGD/R group had condensed nuclei and increased pyroptotic cells, while quercetin and MCC950 treatment significantly reduced nuclear condensation and the number of pyroptotic cells (Figure 2B,C).

### 3.5. Quercetin Reduces the Expression of Pyroptotic Inflammatory Factors

After BV2 cells undergo pyroptosis, a large number of pro-inflammatory factors such as IL-1β, IL-18, and TNF-α are released, while the release of the anti-inflammatory factor TGF-β is reduced. IL-18 and IL-1β play crucial roles in cell pyroptosis. As an inflammatory factor in the brain, TNF-α can enhance the inflammatory response of the central nervous system by affecting the permeability of the blood-brain barrier, thereby invading the central nervous system and causing central nervous system inflammation. ELISA results showed that compared with the Ctrl group, the contents of IL-1β, IL-18, and TNF-α in the OGD/R group were increased; after quercetin treatment, the contents of pro-inflammatory factors were decreased, and its effect was similar to that of the MCC950 inhibitor (Figure 2D–F). The results indicate that quercetin can reduce the expression of inflammatory factors after pyroptosis.

### 3.6. Quercetin Inhibits NLRP3 Inflammasome Activation to Alleviate OGD/R-Induced Pyroptosis of BV2 Cells

The activation of the NLRP3 inflammasome triggers the pyroptosis executor protein GSDMD to execute pyroptosis. Therefore, the activation of the NLRP3 inflammasome is a key factor inducing microglial pyroptosis. Combined with the results of Hoechst/PI staining and GSDMD detection, we can more accurately define the nature of cell death. The increase in PI-positive cells observed in the model group, accompanied by a significant upregulation of GSDMD, suggested that this loss of cell membrane integrity was mainly due to pyroptosis rather than simple late apoptosis. Fluorescence double staining experiments initially verified that quercetin can alleviate microglial pyroptosis, and further research is needed to determine whether quercetin can alleviate OGD/R-induced pyroptosis of BV2 cells by inhibiting NLRP3 inflammasome activation. Western blot results showed that compared with the Ctrl group, the expression of pyroptosis-related proteins NLRP3, Caspase-1, GSDMD, and ASC in the OGD/R group was significantly increased; MCC950 is a specific inhibitor of the NLRP3 inflammasome, which can significantly inhibit the activation of the NLRP3 inflammasome. After treatment with quercetin and MCC950, the expression levels of NLRP3, Caspase-1, GSDMD, and ASC were all significantly decreased (Figure 2G–K). These results indicate that quercetin regulates the pyroptosis axis by inhibiting NLRP3 inflammasome activation, thereby alleviating OGD/R-induced cell pyroptosis.

### 3.7. Quercetin Improves Motor Dysfunction and Neuronal Injury in PT Mice

**Figure 3:**
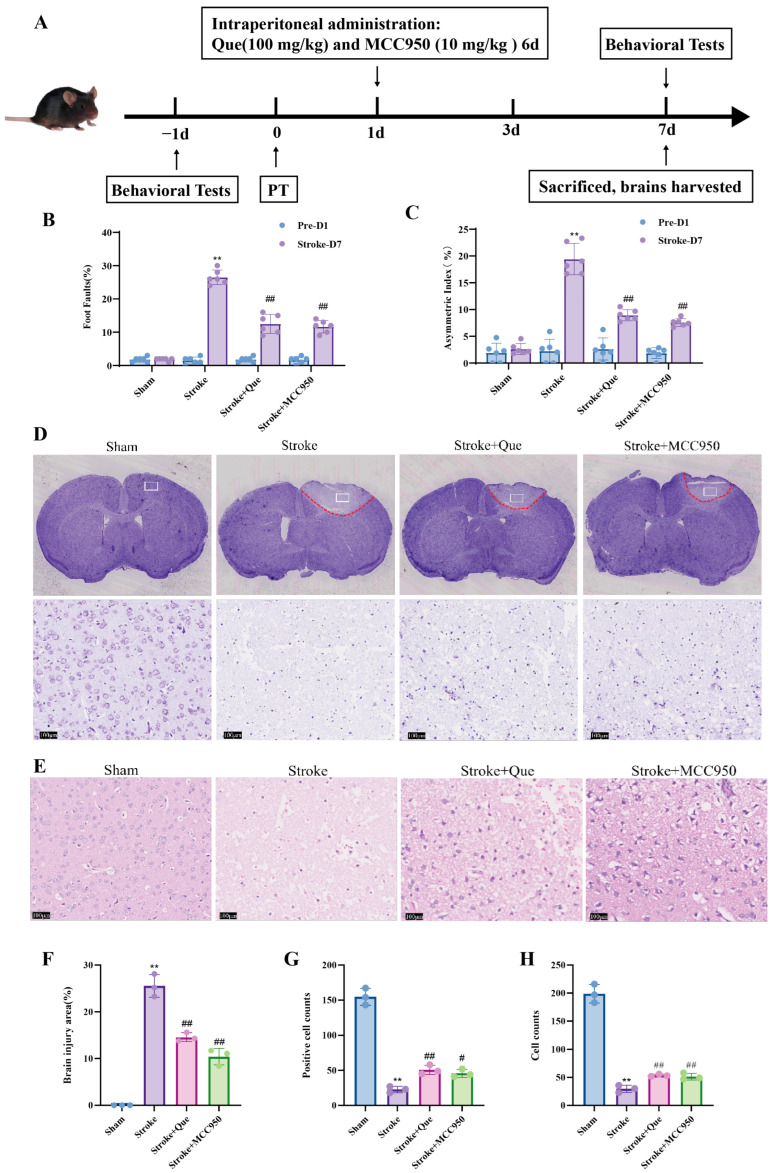
Quercetin improves motor dysfunction and neuronal injury in PT mice. (**A**) Schematic diagram of the time course of in vivo experiments in PT mice. (**B**) Error step rate of mice in grid test 1 day before modeling and 7 days after administration (*n* = 6). (**C**) Forelimb asymmetry rate of mice in cylinder test 1 day before modeling and 7 days after administration (*n* = 6). (**D**) Nissl staining of brain tissue sections in each group, Scale bar = 100 μm; (**E**) HE staining of brain tissue sections in each group, Scale bar = 100 μm. (**F**) Quantitative analysis of brain injury area (*n* = 3). (**G**) Quantitative analysis of Nissl bodies (*n* = 3). (**H**) Quantitative analysis of neurons by HE staining (*n* = 3). Compared with the Sham group, ** *p* < 0.01; compared with Stroke group, # *p* < 0.05, ## *p* < 0.01; Mean ± SD, *n* ≥ 3.

To verify the protective effect of quercetin on motor function injury in mice with cerebral ischemia, the motor cortex of mice was selected as the target of photothrombotic modeling, and injury in this area directly leads to abnormal motor function in mice. Therefore, the recovery of motor function was evaluated by grid test and cylinder test, and the neuronal injury and inflammatory level in the penumbra of the cerebral cortex in this area were detected at the same time. The results showed that compared with before modeling, the error step rate and forelimb asymmetry rate in the Stroke group were significantly increased, indicating that the mice had motor dysfunction after cerebral ischemia; after treatment with quercetin and MCC950, the error step rate and forelimb asymmetry rate were significantly decreased (Figure 3B,C). The results indicate that quercetin can alleviate motor dysfunction in PT mice. Nissl staining results showed that compared with the Sham group, the number of Nissl bodies in the Stroke group was significantly reduced, distributed sparsely, and the damaged area of the motor cortex was enlarged; after treatment with quercetin and MCC950, the number of Nissl bodies was increased, and the damaged area of the motor cortex was reduced (Figure 3D,F). HE staining results showed that the neurons in the Sham group had tight arrangement and clear cell structure, while the neurons in the Stroke group had pyknotic and deeply stained nuclei, increased intercellular spaces, and cavitation; quercetin and MCC950 treatment alleviated neuronal injury (Figure 3E). The above results indicate that quercetin can improve post-ischemic brain injury and exert a protective effect on neurons.

### 3.8. Quercetin Inhibits Inflammasome Activation to Alleviate PT-Induced Pyroptosis

**Figure 4:**
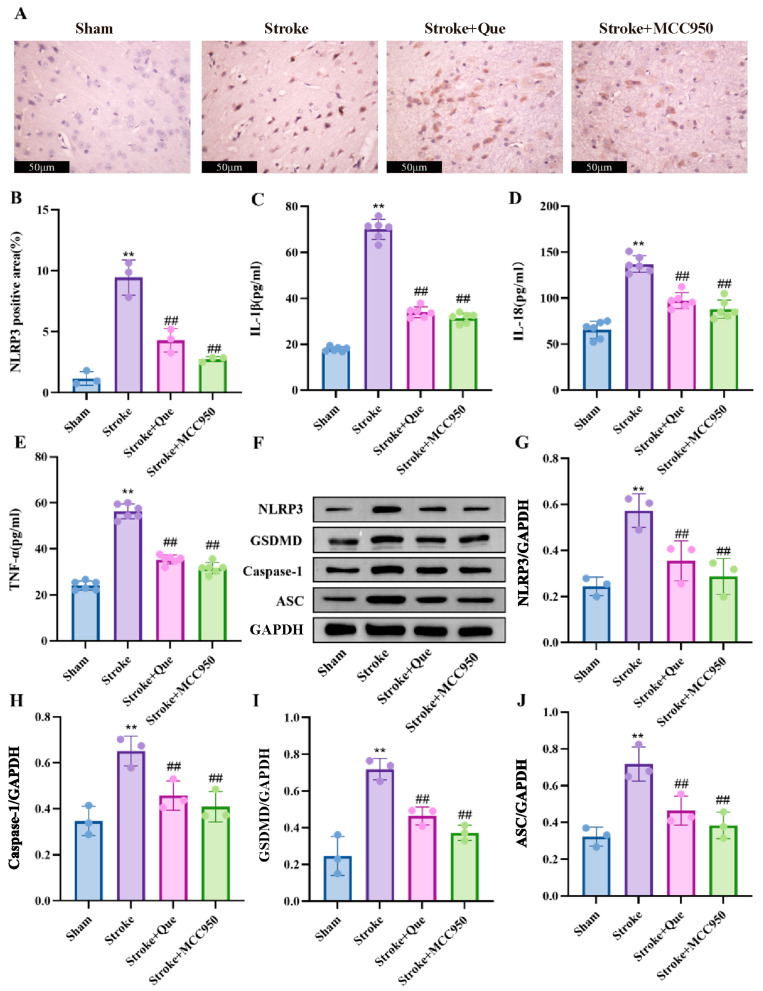
Quercetin inhibits pyroptosis in PT mice by suppressing the NLRP3 inflammasome/pyroptosis axis. (**A**) Immunohistochemical detection of the expression of NLRP3-positive cells in the motor cortex of mice in each group by quercetin, Scale bar = 50 μm. (**B**) Quantitative analysis of NLRP3-positive cells (*n* = 3). (**C**–**E**) ELISA for the expression levels of IL-1β, IL-18 and TNF-α in mouse serum (*n* = 6). (**F**) Representative images of immunoblot analysis of pyroptosis-related proteins in the motor cortex of mice in each group. (**G**–**J**) Quantitative analysis of the expression levels of pyroptosis-related proteins (*n* = 3). Compared with the Sham group, ** *p* < 0.01; compared with Stroke group, ## *p* < 0.01; Mean ± SD, *n* ≥ 3.

The activation of the NLRP3 inflammasome is the primary cause of regulating the pyroptosis axis and triggering the inflammatory response. Therefore, immunohistochemistry was used in this study to detect the expression of NLRP3, a key protein for inflammasome activation, in the motor cortex. Results showed that compared with the Sham group, the number of NLRP3-positive cells in the motor cortex of the Stroke group was significantly increased; compared with the Stroke group, the number of NLRP3-positive cells in the Stroke+Que group and the Stroke+MCC950 group was decreased (Figure 4A,B). After pyroptosis, inflammatory factors such as IL-1β, IL-18 and TNF-α are rapidly released to the outside of the cell through the cell membrane pores, triggering an inflammatory cascade. ELISA results showed that compared with the Sham group, the expression of IL-1β, IL-18 and TNF-α in the motor cortex of the Stroke group was significantly increased; compared with the Stroke group, the expression levels of the above inflammatory factors in the Stroke+Que group and the Stroke+MCC950 group were significantly decreased (Figure 4C,D). Western Blot results showed that compared with the Sham group, the expression levels of pyroptosis-related proteins NLRP3, Caspase-1, GSDMD and ASC in the motor cortex of the Stroke group were significantly increased; compared with the Stroke group, the expression levels of the above proteins in the Stroke+Que group and the Stroke+MCC950 group were significantly decreased (Figure 4F–J). The above data suggested that quercetin alleviates the expression of pyroptosis-related proteins and inflammatory factors in PT mice by inhibiting the NLRP3 inflammasome/pyroptosis axis.

### 3.9. Quercetin Binds Specifically to NLRP3 Protein

Molecular docking simulation technology can effectively predict the binding mode and affinity between small molecules and target proteins, thereby evaluating the potential interaction and selectivity of candidate compounds [26]. To explore the binding effect between quercetin and the NLRP3 protein, AutoDock Vina software was used for docking analysis in this study. The grid box parameters of the NLRP3 receptor in the docking system were set as (x: 21.763, y: 14.444, z: 9.778), and the grid size was (x: 69.75, y: 53.389, z: 54.25). The docking results showed that the optimal binding mode (mode 1) of quercetin and NLRP3 had a binding free energy of −5.299 kcal/mol, which was lower than the reported active inhibitor threshold (−7.0 kcal/mol) [27], suggesting that quercetin has good binding activity and affinity with NLRP3.

**Figure 5:**
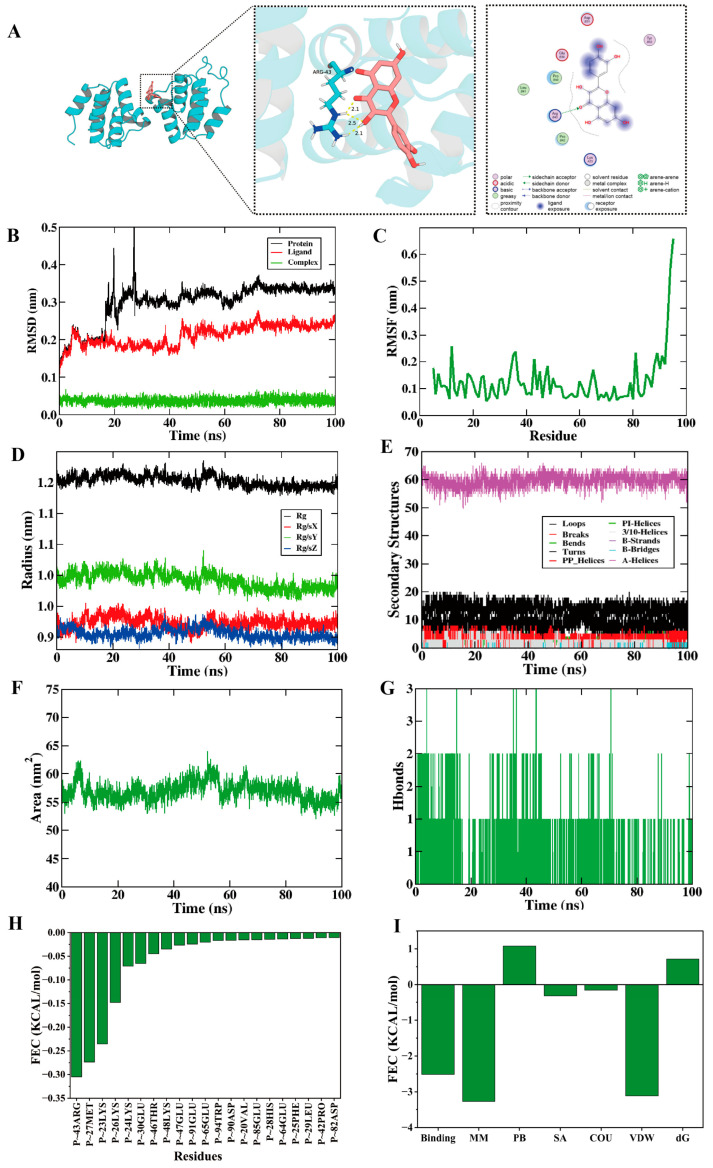
Molecular docking and molecular dynamics simulation characterization of the quercetin-NLRP3 complex. (**A**) Molecular docking of quercetin with NLRP3. (**B**) Root mean square deviation (RMSD) of NLRP3 (black), quercetin (red), and the complex (green) during the 100 ns simulation. (**C**) Root mean square fluctuation (RMSF) of the complex, reflecting residue flexibility. (**E**) Dynamic changes in the secondary structure of NLRP3 (loop: purple; α-helix: black, etc.). (**D**) Radius of gyration (Rg) of the complex (total Rg: black; axis-specific Rg: colored curves). (**F**) Solvent-accessible surface area (SASA) of the complex. (**G**) Changes in the number of hydrogen bonds between quercetin and NLRP3 over time. (**H**,**I**) MM-PBSA energy analysis results derived from molecular dynamics simulations.

As shown in Figure 5A, the binding interface between quercetin and NLRP3 involves multiple key residues: the ARG-43 residue forms interactions with quercetin at distances of 2.1 Å and 2.5 Å, and the binding region also includes residues such as Asp-831, Glu-830, Tyr-832, and Pro-840, which participate in stabilizing the binding conformation of quercetin and NLRP3 through hydrogen bonds, hydrophobic interactions, and other ways.

### 3.10. The Complex Formed by Quercetin and NLRP3 Protein Has Good Structural Stability

To verify the molecular mechanism by which quercetin improves cerebral ischemic injury by targeting NLRP3, multi-dimensional stability and interaction analysis of the complex formed by quercetin and the NLRP3 protein was performed through molecular dynamics simulation in this study. The root mean square deviation (RMSD) results showed (Figure 5B) that the quercetin-NLRP3 complex system (green curve) tended to be stable after approximately 20 ns and entered a balanced state, with the final RMSD stabilized around 0.05 nm, indicating that after quercetin binds to NLRP3, the overall conformation of the complex can maintain good stability, laying a structural foundation for subsequent targeted regulatory effects. The root mean square fluctuation (RMSF) analysis (Figure 5C) showed that the RMSF values of the complex were mostly below 0.23 nm, with low overall residue flexibility, suggesting that the local structural fluctuation of the NLRP3 protein was small after quercetin binding, further confirming the structural stability of the complex. The radius of gyration (Rg) results (Figure 5D) showed that the total Rg was stable around 1.2 nm with small fluctuation amplitude, indicating that the overall structural compactness of the NLRP3 protein remained stable during the 0–100 ns simulation, without obvious expansion or contraction, and quercetin binding did not damage the integrity of the protein core structure. The secondary structure analysis (Figure 5E) showed that the number and proportion of core secondary structures of NLRP3, such as α-helices and random coils, remained basically stable throughout the simulation, with only local dynamic adjustments in secondary structures, indicating that the protein secondary structure framework did not undergo significant folding or unfolding, providing a guarantee for its functional stability.

The solvent-accessible surface area (SASA) analysis (Figure 5F) showed that the SASA of the complex fluctuated significantly during the simulation, suggesting that the surface exposure of the complex changed dynamically after quercetin bound to NLRP3, but did not cause significant disturbance to the overall structure of the protein. The hydrogen bond interaction analysis (Figure 5G) showed that the number of hydrogen bonds between the two fluctuated mostly between 0 and 2, and only 1 hydrogen bond was maintained in most cases, indicating that hydrogen bonds were not the main driving force for the binding between the two. The MM-PBSA energy analysis results showed (Figure 5H,I) that the per-residue free energy decomposition indicated that most residues contributed negatively to the binding free energy, which was beneficial to the binding; the energy component decomposition showed that van der Waals interactions were the main favorable contribution to the binding, the contributions of polar and non-polar solvation energies were close to 0, and the overall binding free energy was close to 0 kcal/mol, indicating that quercetin had basic binding ability but weak affinity with NLRP3, and “hotspot residues” such as P-43ARG and P-27MET dominated the binding specificity. In summary, the molecular dynamics simulation results verified that quercetin can stably bind to the NLRP3 protein, and exert a targeted effect by maintaining the structural stability of the NLRP3 protein and forming a specific binding mode, providing molecular-level support for the mechanism by which quercetin improves cerebral ischemic injury through regulating NLRP3.

## 4. Discussion

It has been reported in the literature that cerebral ischemia can lead to increased vascular permeability, the formation of vasogenic brain edema, and then induce infarction and neurobehavioral abnormalities [28]. Yang Qingli et al. [29] found that quercetin could improve neurological function injury and protect neurons in neonatal rats with hypoxic-ischemic brain injury. Through behavioral analysis, HE staining and Nissl staining, this experiment further confirmed that quercetin treatment could significantly reduce the grid error step rate and cylinder asymmetry rate of mice with cerebral ischemia, increase the number of Nissl bodies in the brain injury area, and alleviate abnormal neuronal morphology, which was consistent with the conclusions of the above studies, and verified the neuroprotective effect of quercetin on cerebral ischemia at the overall level. This study found that quercetin could significantly reduce the cortical injury area after cerebral ischemia, and its core mechanisms of regulating the scope of cerebral ischemic injury and exerting neuroprotection are reflected in three aspects: first, inhibiting the neuroinflammatory cascade, quercetin blocks the signal transduction of the NLRP3/Caspase-1/GSDMD pyroptosis axis by directly targeting and binding to NLRP3 protein and inhibiting its activation, reduces microglial pyroptosis and the release of downstream pro-inflammatory factors such as IL-1β, IL-18 and TNF-α, thereby alleviating secondary neuronal injury mediated by inflammatory factors; second, improving the oxidative stress state after cerebral ischemia, quercetin can effectively scavenge excessive ROS induced by mitochondrial stress, block the activation of the NLRP3 inflammasome from the upstream, and reduce neuronal apoptosis and pyroptosis mediated by oxidative stress; third, maintaining the integrity of the structure and function of the blood-brain barrier, existing studies have confirmed [12] that quercetin can upregulate the expression of tight junction proteins in brain tissue, inhibit the increase in blood-brain barrier permeability, and reduce the infiltration of peripheral inflammatory cells into the brain.

This study selected the mouse motor cortex as the target of the photothrombotic model. As the core center for regulating limb movement, the NLRP3 inflammasome activation and cell pyroptosis response mediated by microglia are more severe after ischemia in this area [30]. The study found that quercetin could effectively inhibit the activation of the NLRP3/Caspase-1/GSDMD pyroptosis axis in the ischemic motor cortex, and its anti-inflammatory effect was positively correlated with the improvement of motor function in mice, indicating that the neuroprotective effect of quercetin has brain region targeting, which provides a theoretical basis for the clinical treatment of ischemic stroke with motor dysfunction as the main symptom.

As an atypical form of programmed cell death under inflammatory conditions, the release of LDH and inflammatory factors such as IL-1β and IL-18 during pyroptosis is a key link exacerbating cerebral ischemic injury [26,27,28]. In this study, LDH release assay and Hoechst33342/PI staining results showed that quercetin can significantly reduce the LDH release and PI-positive cell rate of BV2 cells after OGD/R, indicating that quercetin can directly inhibit microglial pyroptosis. Combined with ELISA and Western Blot results, quercetin simultaneously downregulated the expression of pyroptosis-related inflammatory factors and NLRP3, Caspase-1, ASC, GSDMD proteins, and its effect was similar to that of the specific NLRP3 inhibitor MCC950, suggesting that the inhibitory effect of quercetin on the NLRP3 inflammasome/pyroptosis axis is one of the core mechanisms for its neuroprotective effect.

Pyroptosis is an atypical form of programmed cell death under inflammatory conditions, and LDH as well as inflammatory factors such as IL-1β and IL-18 released by cell membrane rupture during this process are key links exacerbating cerebral ischemic injury [31,32,33]. In the present study, results of the LDH release assay and Hoechst33342/PI double staining showed that quercetin could significantly decrease the LDH release and the rate of PI-positive cells in BV2 cells after OGD/R. Combined with the results of ELISA and Western Blot, quercetin simultaneously downregulated the expression of pyroptosis-related inflammatory factors and NLRP3, Caspase-1, ASC and GSDMD proteins, with an effect similar to that of MCC950, a specific inhibitor of NLRP3. These findings suggest that the inhibitory effect of quercetin on the NLRP3 inflammasome/pyroptosis axis is one of the core mechanisms underlying its neuroprotective effects. The levels of IL-1β, IL-18 and TNF-α in serum were detected in this study, and the results showed that their changing trends were highly consistent with those of pyroptosis markers in the brain. Accumulating studies have shown that when severe neuroinflammation or blood-brain barrier damage occurs, inflammatory factors such as IL-1β and IL-18 produced in the central nervous system can enter the peripheral blood circulation through the damaged blood-brain barrier [34]. Therefore, the elevation of these cytokines in serum can serve as an indirect and effective indicator of the intensity of inflammatory and pyroptotic activity in the brain, reflecting the “spillover effect” of local cerebral pyroptosis on the systemic system. Meanwhile, the detection of serum inflammatory factors and the indexes of brain proteins such as NLRP3 and Caspase-1 mutually corroborate each other, further supporting the conclusion that brain tissue is the source of inflammatory initiation. The rationality of this detection method is also supported by a large number of literatures: in stroke research, multiple studies have confirmed that the serum levels of IL-1β, IL-18 and TNF-α are closely related to the degree of brain injury and neurological deficit [35,36,37]. A recent meta-analysis including 26 studies with a total of 6573 patients with acute stroke showed that the serum levels of IL-1β, IL-18 and TNF-α were significantly elevated and correlated with the prognosis of patients [36]; in animal studies, Noori et al. also successfully evaluated the activity of central inflammasomes by detecting changes in serum inflammatory factors in a rat model of spinal cord injury [34].

In the pathological process of cerebral ischemia, ROS acts as a key agonist of NLRP3 inflammasome activation, and its excessive production can induce inflammatory cascade reaction and cell pyroptosis [38,39]. Huang et al. [40] reported that quercetin exerts a brain protective effect by alleviating oxidative stress, and the present study further confirmed that quercetin can significantly reduce the production of ROS in BV2 cells after OGD/R, providing upstream mechanistic support for its inhibition of the pyroptosis axis.

Although cellular and animal experiments have confirmed the inhibitory effect of quercetin on the NLRP3 inflammasome/pyroptosis axis, direct molecular evidence for its interaction with NLRP3 protein is lacking. To address this issue, molecular docking and molecular dynamics simulation techniques were introduced in this study. Molecular docking results showed that quercetin could specifically bind to the NACHT domain of NLRP3 protein, with an optimal binding free energy of −5.299 kcal/mol, which is lower than the active inhibitor threshold. The binding interface involves key residues such as ARG-43 and Asp-831; molecular dynamics simulation further verified the structural stability of the quercetin-NLRP3 complex. The RMSD of the complex was basically stable at about 0.05 nm during the 100 ns simulation, and van der Waals interactions were the main driving force for binding. In addition, “hot spot residues” such as P-43ARG and P-27MET were identified, which provides molecular evidence for quercetin directly targeting NLRP3 protein at the atomic level.

From a translational perspective, this finding provides an important design idea for the development of NLRP3-targeted drugs against ischemic brain injury from natural products: as a natural flavonoid widely present in fruits and vegetables, quercetin has the advantages of low toxicity and high biocompatibility. Its binding site with NLRP3 (the NACHT domain) can be used as a core target for subsequent drug modification. By performing structural modification on the quercetin mother nucleus (such as introducing hydrophilic groups to improve blood-brain barrier penetration and optimizing side chains to enhance the binding force with hot spot residues), it is expected to develop NLRP3-targeted derivatives with high selectivity and high activity, making up for the toxicity and targeting defects of chemically synthesized inhibitors. Meanwhile, the research system of “cellular/animal experiments+molecular docking/MD simulation” established in this study also provides a replicable technical route for the study of the molecular mechanism of other natural products against cerebral ischemia.

A comparison of the binding characteristics and biological activity of quercetin with NLRP3 with those of classic NLRP3 inhibitors can more clearly clarify its advantages and disadvantages: MCC950, a classic small molecule inhibitor, is the most extensively studied specific inhibitor of NLRP3 at present. It can directly bind to the NACHT domain of NLRP3 and inhibit its ATPase activity, with a binding free energy of about −8.5 kcal/mol with NLRP3, which is much lower than −5.299 kcal/mol of quercetin in this study. This suggests that the binding affinity of MCC950 with NLRP3 is significantly higher than that of quercetin, which also explains why MCC950 can achieve a potent inhibitory effect on NLRP3 at a low concentration; another inhibitor OLT1177 blocks its interaction with ASC by binding to the PYD domain of NLRP3, with a binding free energy of about −7.3 kcal/mol, which also has a stronger binding ability than quercetin.

The main innovation of this study is that it is the first to confirm that quercetin may specifically inhibit the activation of the inflammasome-pyroptosis axis in microglia and the release of downstream inflammatory factors by directly binding to the NACHT domain of NLRP3 protein and stabilizing its conformation, thereby alleviating cerebral ischemia-induced inflammatory injury. This finding not only reveals a new target for the neuroprotective effect of quercetin at the molecular level, but also provides direct theoretical basis and design ideas for the development of precise anti-neuroinflammatory drugs targeting NLRP3.

As an exploratory study combining computational biology with in vitro and in vivo experiments, the molecular findings of this study are predictive hypotheses that still need to be further verified by subsequent experimental methods. Limited by the lack of experimentally resolved structures of the NLRP3 NACHT domain, the molecular docking and molecular dynamics simulation in this study only proposed a possible mode of action of quercetin at the atomic level, and did not confirm the direct physical binding between quercetin and NLRP3 protein through in vitro biochemical experiments, nor verify the functionality of the predicted binding sites. To address the above issues, targeted verification experiments will be carried out in the follow-up: (1) The direct physical binding between quercetin and NLRP3 protein can be verified by experiments such as surface plasmon resonance or cellular thermal shift assay. (2) Site-directed mutagenesis technology (e.g., targeting key residues of the Walker A motif in the NACHT domain) can be used to observe whether quercetin still maintains the inhibitory activity on NLRP3 after mutation, so as to reversely verify the predicted binding sites in this study.

This study has confirmed from the molecular, cellular and whole animal levels that quercetin can directly bind to NLRP3 and inhibit its mediated neuroinflammation and pyroptosis. On this basis, future research can be further deepened around the following directions: first, verify the predictive hypothesis proposed in this study that “quercetin may bind to the NACHT domain of NLRP3 protein” through the above in vitro biochemical and molecular biology experiments, and elucidate the inhibitory mechanism of the quercetin-NLRP3 complex on the ternary assembly of inflammasomes by using structural biology methods; second, develop a brain drug delivery system based on NLRP3-targeted delivery to improve the enrichment and efficacy of quercetin in the ischemic motor cortex; third, carry out the design and optimization of quercetin derivatives based on the key binding sites predicted in this study, and promote their development into clinical candidate drugs with high selectivity and high bioavailability.

This study also has certain limitations: first, the number of biological replicates for Western Blot detection was *n* = 3. Although the repeatability of the results was verified by pre-experiments, the sample size is relatively small. For models with high inherent biological variability such as cerebral ischemia-neuroinflammation, the sample size in this study still has the potential problem of insufficient statistical test power; second, this study only focused on ischemic injury in the mouse motor cortex, and did not investigate the protective effect of quercetin on other brain regions such as the hippocampus and striatum after cerebral ischemia. Follow-up studies will carry out multi-brain region analysis to improve the research results; third, although molecular docking and MD simulation in this study revealed the binding mode of quercetin and NLRP3 at the atomic level, computer simulation research has its inherent limitations, making it impossible to completely and accurately predict the actual target binding effect in vivo. This is mainly reflected in that the MD simulation is based on the interaction between the in vitro purified NLRP3 protein monomer and quercetin, without considering the complex intracellular microenvironment in vivo (while NLRP3 exists in the form of multimers in vivo, and its conformation is regulated by the intracellular redox state and protein-protein interactions, all of which will affect the actual binding efficiency of quercetin and NLRP3); fourth, the mechanistic verification of the NLRP3/Caspase-1/IL-1β/IL-18 pyroptosis axis in this study only completed the detection of molecular expression and activation levels, and did not directly verify the functional necessity of downstream IL-1β/IL-18. The causal relationship deduction of the signaling pathway is partially based on existing literatures, and the relevant conclusions are working hypotheses that need to be further verified by subsequent experiments as follows: (1) Pretreat mice with cerebral ischemia model with IL-1β/IL-18 neutralizing antibodies, and observe whether the protective effect of quercetin on cerebral ischemic injury changes after knocking down endogenous IL-1β/IL-18, so as to verify that these factors are the key downstream mediators for quercetin to exert its effects; (2) Use siRNA/shRNA gene silencing technology to knock down the expression of IL-1β/IL-18 in BV2 microglia, and detect whether the regulatory effect of quercetin on cell pyroptosis and inflammatory response in the OGD/R model is weakened, so as to verify the dependence of the protective effect of quercetin on the downregulation of IL-1β/IL-18 at the cellular level; fifth, the present study used Hoechst/PI double staining to evaluate cell death. This method can effectively identify the loss of cell membrane integrity (PI positive), but cannot clearly distinguish pyroptosis from late apoptosis or secondary necrosis at the morphological level. Both pyroptosis and late apoptosis can be PI positive, so the occurrence of pyroptosis cannot be confirmed solely by this staining result. This limitation requires us to make a comprehensive judgment by combining other molecular markers when interpreting the data; sixth, the present study detected IL-1β, IL-18 and TNF-α through serum, which provided important data reflecting the peripheral inflammatory response and was consistent with the changing trend of pyroptosis markers in the brain. However, we acknowledge that the peripheral levels of these cytokines cannot be completely equivalent to the real concentration in the local brain. Future studies can further verify the conclusions of this study by detecting inflammatory factors in cerebrospinal fluid or brain interstitial fluid.

**Figure 6:**
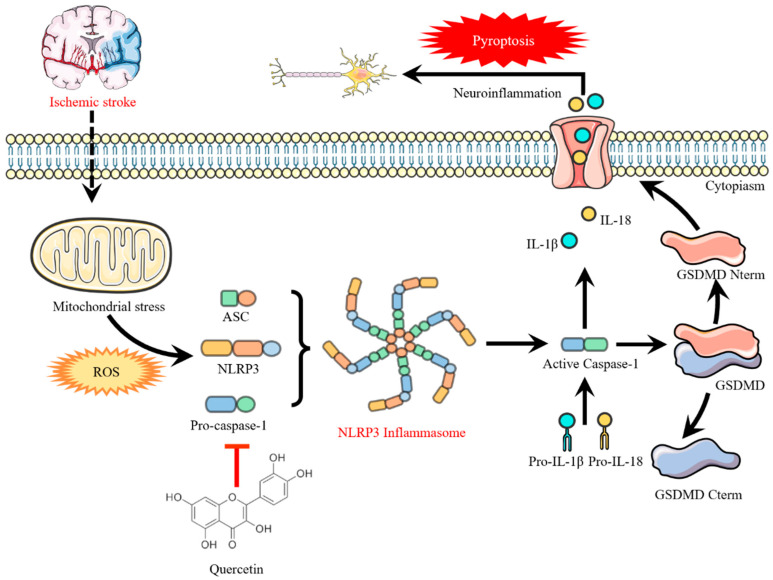
Schematic diagram illustrating the hypothetical mechanism by which quercetin modulates the NLRP3 inflammasome/pyroptosis axis to alleviate BV2 microglial pyroptosis induced by cerebral ischemia. During cerebral ischemia, mitochondrial stress induces the massive production of ROS, which activates the NLRP3 inflammasome (recruiting ASC and Pro-Caspase-1 for assembly); activated Caspase-1 on the one hand cleaves Pro-IL-1β/IL-18 into mature inflammatory factors to trigger neuroinflammation, and on the other hand cleaves GSDMD to generate N-terminal fragments, which form pores in the cell membrane leading to cell pyroptosis and exacerbating neuronal injury. Quercetin scavenges excess ROS, blocks the upstream signal of NLRP3 activation to inhibit the assembly of the NLRP3 inflammasome; ultimately reduces the release of inflammatory factors and cell pyroptosis, and alleviates cerebral ischemic injury.

In summary, this study confirmed through multi-level and multi-technical approaches that quercetin can exert a neuroprotective effect against cerebral ischemic injury by inhibiting the activation of the NLRP3 inflammasome, downregulating Caspase-1 activity and the expression of IL-1β/IL-18, and improving the oxidative stress state; and based on the existing experimental data and published literatures, a hypothesis of quercetin regulating the NLRP3/Caspase-1/GSDMD pyroptosis axis is proposed (Figure 6), and its specific causal regulatory relationship still needs to be further verified by subsequent experiments. The application of molecular docking and molecular dynamics simulation technologies not only provides solid molecular evidence for the mechanism of action of quercetin, but also offers new ideas and experimental basis for the targeted treatment of cerebral ischemic inflammatory response.

Proposed working model: Based on the observed correlation that quercetin inhibits NLRP3 inflammasome activation and reduces downstream CASP1 activity and IL-1β/IL-18 levels, we propose this hypothetical model. Dashed lines indicate relationships that are inferred from established literature but require direct experimental validation in this specific model.

## 5. Conclusions

This study demonstrates that quercetin alleviates cerebral ischemic injury by inhibiting the NLRP3/Caspase-1/GSDMD pyroptosis axis in microglia. Integrating multi-disciplinary approaches, we provide the first evidence that quercetin directly binds to the NACHT domain of NLRP3, suppressing inflammasome activation. These findings establish quercetin as a promising natural lead compound for targeting NLRP3 in ischemic stroke.

## Data Availability

The original contributions presented in this study are included in the article. Further inquiries can be directed to the corresponding author(s).
